# The Impact of Mental Illness Stigma on Psychiatric Emergencies

**DOI:** 10.3389/fpsyt.2020.00573

**Published:** 2020-06-19

**Authors:** Antônio Geraldo da Silva, Leonardo Baldaçara, Daniel A. Cavalcante, Nicoli Abrão Fasanella, Antônio Pacheco Palha

**Affiliations:** ^1^Faculdade de Medicina, Universidade do Porto, Porto, Portugal; ^2^Associação Brasileira de Psiquiatria, Rio de Janeiro, Brazil; ^3^Asociación Psiquiátrica de América Latina; ^4^Medicine, Universidade Federal do Tocantins, Palmas, Brazil; ^5^Department of Psychiatry, Interdisciplinary Laboratory in Clinical Neuroscience (LiNC), Universidade Federal de São Paulo (UNIFESP), São Paulo, Brazil; ^6^GAPi (Early Psychosis Group), Universidade Federal de São Paulo (UNIFESP), São Paulo, Brazil; ^7^Medicine, Pontifícia Universidade Católica de São Paulo, São Paulo, Brazil

**Keywords:** stigma, psychiatric emergencies, stigma-related stress, crisis, intervention

## Abstract

Psychiatric emergencies are severe behavioral changes secondary to worsening mental illness. Such situations present a risk to the patient and other people, so they need immediate therapeutic intervention. They are associated with feelings of fear, anger, prejudice, and even exclusion. The attitudes of professionals and factors related to the workplace culture in health can help to perpetuate stereotypes and interfere with the quality of care. Stigma has undesirable consequences in patients with mental disorders. Certain measures can reduce stigma and provide a more dignified way for patients to recover from the crisis. This article aims to discuss the causes of stigma, ways of dealing with it, and achievements that have been made in psychiatric emergency care settings.

## Introduction

According to Link and Plan (2001), Erving Goffman’s book *Stigma: Notes on the Management of Spoiled Identity* (1963) stimulated the expansion of research on the causes and consequences of stigma (1). Among the many current definitions of stigma, we can extract that stigma exists when the effect of trivializing, labels, loss of status, and segregation happen at the same time in the same situation (1).

Although the quality and effectiveness of mental health treatment and services have improved greatly over the past 50 years, therapeutic revolutions in psychiatry have not yet been able to reduce its stigma (2). Mental illness-related stigma, including that which exists in the healthcare system and among healthcare providers, has been identified as a major barrier to treatment and recovery, resulting in poorer care quality for mentally ill people (3, 4). Stigma also impacts the treatment-seeking behavior of health providers themselves and negatively mediates their work environment (4, 5).

Psychiatric emergencies are severe behavioral changes secondary to worsening mental illness. Such situations present a risk to the patient and other people, so they need immediate therapeutic intervention (6, 7). Although such emergencies can also be secondary to physical illnesses, what differs them from other emergencies is precisely the presence of severe behavioral changes.

In most cases, they represent extreme severity in mental illness, they are associated with feelings of fear, anger, prejudice, and even exclusion. Among the most prevalent psychiatric emergencies are suicidal behavior, severe depressive or manic episodes, self-mutilation, severely impaired judgment, severe self-neglect, substance intoxication or abstinence crises, and aggressive agitation (6, 7). Adequate management of such situations can reduce patient suffering and prevent the perpetuation of stigma.

This article aims to discuss the causes of stigma, ways of dealing with it, and achievements that have been made in psychiatric emergency care settings. Although there are different models of care for psychiatric emergencies, we will consider situations whose general management principles are the same in different environments.

## Search Strategy and Selection Criteria

This Review of the management of Psychiatric Emergencies in situations of public calamity. The strategy was used to search the following international electronic databases; Pubmed (1990–present), Scielo (1990–present), and Cochrane Database of Systematic Reviews (1990–present). The search terms comprised: psychiatric emergencies, emergencies, mental disorders, calamity, disasters, epidemic, and pandemic. We supplemented the search results with important publications.

## Causes of Stigma for Psychiatric Disorders

Stigma stems from several sources (personal, social, or family) that work synergistically and can cause several complications throughout life (2, 8). Studies have shown that the stigma of mental illness can stem from a lack of understanding or information, as well as from the meaning of the illness itself, such as strange behavior or agitation (2, 9). Since no specific study has been conducted on stigma in psychiatric emergencies, we will assess some general hypotheses about mental illness stigma and apply them to emergency situations, regardless of where they are treated.

### Violence as a Possibility

Agitation without or with aggressive behavior is common in situations of psychiatric emergencies. However, in this case, the aggressiveness or state of violence must be seen as a complication of mental illness. As expressed by one author, there is a subjective and cultural relationship between mental illness and violence, and this perception has implications for patients, with an increased sense of isolation secondary to discrimination (10).

One study found that 61% of adults believed that an individual with schizophrenia was somehow likely to be violent towards others (11). On the other hand, a 2009 study concluded that mental illness singly does not predict violent behavior (12). Although the analyses showed that aggressive agitation does occur in people with severe mental illness, its occurrence is only significant in those with co-occurring substance abuse and/or dependence. Thus, albeit a minority of patients have addiction issues, it is a mistake to generalize the possibility of aggression to any diagnosis or any potential emergency.

Psychomotor agitation may or may not be associated with aggressiveness. Although it does occur in a small percentage of people with mental disorders, psychiatric emergencies can trigger agitation while simultaneously compromising the patient’s autonomy. Agitation and bizarre behavior are stereotypes created about people with mental illness, and these intensify when a patient has a crisis.

Finally, little is known about the risk and causal factors related to social and contextual issues of aggression, although literacy reports that mental patients are more often victims than perpetrators of violence (13). People with mental illness should be protected, and in the context of psychiatric emergencies, how they are handled is of critical importance.

### Shame

People can take a long time to seek treatment and hide their symptoms, or when they become apparent, the family hides them at home or sends them to a distant hospital. This reaction is very similar to that of some families who try to hide when a relative committed suicide (9). Attempting to hide symptoms can impede treatment seeking and lead to worsening of the condition. More immediate services, such as outpatient clinics, community services, and even emergency units can make patients feel exposed and assume the presence of a disease.

Parents of patients with mental illnesses have a greater sense of stigma, in particular embarrassment and shame (14). In turn, shame in the family of people with mental illness may be greater than that of families of people with cancer (15).

One study states that the real prevalence of psychiatric emergencies may be higher than that observed, and therefore, patients may take a long time to seek care for fear of stigma and the high cost of psychiatric treatment (16). Another recent study investigated motivating factors for seeking treatment in Lebanon and found that relatively few mentally ill patients (19.3%) seek treatment. In higher education, the highest income and the female sex were identified as positive predictors for the demand for treatment, and the recognition of than the importance and impact of mental illness (16, 17), especially in emergency situations.

### Lack of Knowledge

While parts of the population are aware of mental illness, others are still unaware of some diagnoses, their causes, and their impact. For example, a study in China showed that 60% of respondents think that external stressors originate mental illness and only 31.7% knew about International Suicide Prevention Day (18). Evidence suggests that factors that influence avoiding or postponing treatment include ignorance about aspects of mental illness and discrimination (19).

Many seek to learn about mental illness in the mass media, including movies and social networks. What they see, of course, shapes the way they think about both mental illness in general and individual disorders. Unfortunately, the media’s portrayal of mental illness is largely negative and/or inaccurate, which makes social stigma especially problematic. Since psychotic breaks, suicide, and aggravating situations can be shown in distorted or even comical ways, the severity of emergency situations involving mental illness may not be recognized.

Without proper information from family members, even the patients themselves may have difficulty recognizing a worsening of their condition, and when they do, they may have difficulty deciding where to look for help. For example, reluctance engaging suicidal people is often based on a fear of exacerbating their condition and further stimulating suicidal behavior. However, studies have shown that this fear is unfounded, and conversations about suicidal tendencies can even reduce symptoms (20, 21). The recognition that suicidal behavior is serious and that treatment seeking often requires help makes it easier for family members and other caregivers to bring in patients for emergency treatment before a fatality can occur.

### Negative Attitudes

Distorted ideas about mental illness also contribute to the perpetuation of negative and inaccurate images. Public research has shown that eating disorders and alcohol and drug abuse are often seen as caused and maintained by the patient’s own (22–24). The belief that substance abuse is due to one’s own choices and attitudes can influence the value and adequacy of public alcohol and drug services and treatments (24). People with these disorders, as well as individuals with anxiety and depression, are seen as just needing to change their habits and calm down. Such views are rarely understood as diseases, such as cancer or heart disease (23). Emergency situations, such as severe dependence, withdrawal syndrome, delirium, and induced psychosis may fail to be correctly addressed due to stigma. In addition, the perception that patients will be treated negatively can cause them or even family members to avoid seeking treatment.

Among other distorted views are the idea that mental health problems are not common, that people affected are unlikely to recover and that treatment does not bring results (23). It is dangerous to think that mental illness treatment is ineffective, since it can lead people to ignore emergency situations and fail to seek help for them.

Another result of stigma is social distancing from individuals with mental illness. Social distance regarding individuals with mental illness has been measured in some situations (at work, among neighbors, and in marriage) (25). Distancing can lead to difficulties in recognizing emergencies, as well as in offering support, especially for patients who cannot understand their own situation. This problem can be even more serious when social distancing occurs within the family itself.

Finally, a situation may be even more serious: structural discrimination. Where, for example, setting up treatment sites for mental illness in distant areas, many of which are difficult to access, can give the feeling that the problem is not in your region. Another consequence of the distance is being able to transmit the idea of being in poor places in the city, full of crimes and dangerous, which can produce even more false beliefs and negative reactions (1). As a consequence, people with mental illness are much more likely to be victimized.

### The Impact of Therapeutics

Patients may associate treatment for mental illness, whether on an outpatient basis, in hospital wards or in intensive care, with fear, distorted beliefs and even negative memories from prior experience. Despite taking their medication regularly, 25–50% of patients do not report beneficial changes (26, 27) or feel that treatment as something coercive (27, 28), which often leads them to discontinue their medication (40–70% of patients) (27, 29).

When patients are referred for emergency treatment, they may arrive voluntarily or involuntarily. The first approach involves empathy and verbal persuasion, but when life is at risk, whether the patients’ or those around them, they must be bought in, even if against their will. Regardless of whether the symptoms are in remission by the end of treatment, the entire process may be remembered with sadness, resentment, and shame. Patients may be placed in an environment with unknown people (including professionals) and may not be treated according to their wishes (often with injectable medications). However, even with voluntary admission, patients can be treated in a place they are ashamed of or have contact with other patients with similar or worse conditions.

Psychotropic medications can also be seen as stigmatizing, and in an emergency environment, patients are forced to use them, often at higher doses and frequencies than in outpatient treatment. Some medications can cause unpleasant side effects and give patients the feeling that they are no longer in control of their life.

Physical restraint, which is also associated with emergency psychiatric treatment, worsens the stigma for patients and consequently influences adherence to medical treatment (30, 31). Training the healthcare teams to use restraint correctly and only as a last resort is essential, although not all teams are prepared for this.

### The Attitudes and Beliefs of Health Professionals

Research has point some issues out that contribute to stigma in healthcare, either directly or indirectly impacting access to care and care quality for people with mental illnesses (4). Since emergency cases require immediate assistance, healthcare professionals must be prepared to deal with aggravated mental illness and any related stigma (4).

In Brazil, psychiatrists also presented negative ideas about people with schizophrenia. Negative stereotypes were present along with the belief that tolerance to side effects of psychotropic medications. Already, being older was related to less prejudice (32). Lauber et al. (33) found that, in private practice, psychiatrists commonly stereotype individuals with psychiatric disorders and that stigmatizing actions were not different from those of the general population (32). In another study carried out with mental health professionals in hospitals, the same authors observed that some maintain more social distance from individuals with schizophrenia than other people (32, 34). Rettenbacher et al. (35) suggested that psychotropics could be a source of stigma, reporting that almost all psychiatrists evaluated considered psychopharmacotherapy important, but only 71.4% of them followed the same treatment if they are diagnosed with schizophrenia (32). This type of attitude can exacerbate emergencies, bearing in mind that many patients require involuntary intervention and are given higher doses and frequencies of medication over a few hours or days.

The attitudes of professionals and the culture of the workplace can help to sustain stereotypes and hinder care. The nature of contact in healthcare environments, especially the in the emergency room and the psychiatric emergency unit, it makes professionals keep in touch with people who have severe and chronic symptoms. This could, paradoxically, perpetuate rather than dispel stereotypical beliefs (3). What’s more, the connection is usually biased due to the inherent imbalance of power between healthcare professionals and patients, which could mitigate any positive effects of contact (3, 36–38). However, the undesirable effects of contact seem to decrease among professionals with greater experience and age, again demonstrating that experience is a factor that can reduce stigma and inexperience a factor of perpetuation (3, 32). In psychiatric emergencies, whether treated on an outpatient basis, in an infirmary, or in emergency care units, the team’s experience, combined with their treatment approach, can reduce negative and stigmatizing attitudes and result in better patient support.

Rossler (39) reported several variables of the work environment that restrict care quality and can eventually lead to professional burnout, counting non-supportive environments, unsupported locations, restricted means, insufficient facilities, and stigma towards the mental health team. Health professionals reported that these unfavorable conditions impaired the quality of care provided (40, 41). For instance, nurses reported that poor availability of resources and infrastructure hampered security (locations that handle psychiatric emergencies must provide adequate protection for patients and staff), which exacerbates the insecurity of caring for patients with mental illness and may delay or to keep away from care (41, 42).

On the other hand, professionals who care for people with substance use disorders, who have better support in their services, showed more positive attitudes towards patients (40). Experience and age were also related to lower burnout (3), while younger psychiatrists are prone to work-related stress and burnout symptoms (39). Other causes of stress include a lack of positive feedback, poor pay, and an unpleasant workplace. A patient’s suicide is also an important variable for many professionals, and many report symptoms of post-traumatic stress after an event (39).

Stigma can also affect health professionals, which could exacerbate public stigma as well as influence postures toward look up treatment. In a review of the topic, Sartorius et al. (43) investigated the way psychiatrists and psychiatry are viewed by different groups. Public opinion often has the idea that psychiatry does not produce results and can even be harmful, and that psychiatrists are low-status doctors who use too many psychotropic medications. The media presents psychiatry as a specialty without training, representing psychiatrists as madmen, healers, or even charlatans. Medical students and other medical fields view psychiatry in a low light, which discourages many students from pursuing a career in mental health (42, 44).

Other investigations found that health professionals ought to be more familiar with the cultural and racial differences of patients with mental illness (45, 46) and, although many of these professionals believe that training for such information is important, it is rarely offered in health services (46). Differences in the way of expressing oneself, health professional’s misunderstandings about cultural beliefs about mental illness and its treatment are among these factors (42, 45, 46). Greater awareness of cultural norms about mental illness among health professionals could increase treatment seeking by minorities in psychiatric emergencies and lead to better care (42, 46). Therefore, there is an urgent need for training to prepare health professionals for patients with psychiatric emergencies.

### Conclusions About Causes of Stigma in Psychiatric Emergencies

We found few studies on the causes of stigma for mental illness, even less that referred to situations of psychiatric emergencies. The majority are publications of non-systematic or narrative reviews that demonstrate the opinion of the author himself rather than evidence actually observed. There are some cross-sectional studies and very few longitudinal studies that observe stigma in mental illness, but not in emergencies. In addition, many present such data indirectly, that is, they were not studies that aimed to assess stigma and this data appeared in a secondary way. Thus, there is still a lack of studies that assess quantitatively and impartially the cause and effect relationship of factors that may be related to stigma, especially in emergencies. The items presented above are theories and should be investigated in future research.

## Consequences of Stigma in Emergency Care Access

Stigma can conduct to very negative consequences in patients with mental illness. Studies have shown that when labeled as “schizophrenic,” patients feel a change in the way they are treated (47). This label affects the way patients interact with healthcare services as well as the world, since the illness becomes the central aspect of the patient’s identity (48). The impact of stigma on patient self-esteem should also be mentioned, since it is associated with a higher risk of depression and suicide (49).

### Reduced Social Support and Treatment Seeking

The stigma of mental illness can also lead to reduced social support for patients, since it results in social isolation and impedes their reintegration into society (50), which can prolong stays in emergency services and specialized wards. Another effect of stigmatization is the fear of being discriminated against in psychiatric treatment centers, especially in emergency settings, environments which can involve negative stereotyping. Stigma can, thus, create barriers between patients and health services, resulting in reluctance to seek treatment (51).

Another problem is that patients or their families may delay treatment seeking during a psychiatric emergency because they do not wish to be in or have their relatives put in such places (3). Consequently, patients may arrive at the emergency unit in a more severe state, where no mental health services may be available or where those have failed, which creates further difficulties for staff.

### Reduced Investment in Emergency Care

Stigma may also impact the financial and political support received by psychiatric services (52, 53). Over the past few decades, there has been a progressive reduction in psychiatric beds worldwide (53), in part due to a lack of government interest, since spending money in psychiatric beds is not popular. Without strong political support, severely mentally ill patients may have not only poorer psychiatric assistance but also poorer health outcomes, as well, such as premature mortality from preventable diseases (52, 54, 55). Thus, better resource allocation throughout the health system is needed to achieve better access to inpatient care for physical and mental health conditions. In this context, emergency care is hampered by a lack of investment, since psychiatric emergency units require expenditures for facilities and trained staff.

A lack of psychiatric beds could lead to lower care standards, since staff would be working in a high-stress environment with limited resources to adequately manage severely ill patients. The situation is even more problematic in psychiatric emergency settings, since transferring patients to inpatient units is becoming increasingly difficult due to bed closures. As a result, emergency services become even more crowded and hostile, contributing to stigma. Overloaded emergency services, together with patient severity, also perpetuate an image of mentally ill people in crisis.

### Negative Image of Mental Illness

Some psychiatric disorders may involve aggressive and unpredictable behavior, especially psychotic disorders. Such crises could contribute to the misinterpretation that psychiatric patients are dangerous and should be isolated from society. Often patients in crisis are taken from their homes to psychiatric emergency units by the police rather than medical emergency teams, which reinforces a negative image.

Medication also plays a role in the stigma debate. Adverse effects can lead to treatment non-adherence, increasing the risk of psychotic breaks. On the other hand, new drugs with fewer side effects have already proven effective in decreasing the risk of relapse, suicide, and re-hospitalization (29). However, most of these drugs are not easily accessible due to their higher costs. This results in a scenario where the underprivileged have fewer opportunities to stabilize their illness, making them even more vulnerable to negative outcomes. The use of certain psychotropic medications, especially more traditional ones with visible side effects, helps worsen the stigmatic image of patients. Many public emergency services only provide the cheapest available medications, which are not always the best option.

### Haste

Some patients have more frequent psychotic breaks and need emergency assistance more often. Due to the previously mentioned bed-closure problem, some of these patients are discharged prematurely and can be left without community alternatives. As a result, the high complication rate for severely mentally ill patients may also contribute to stigma. Inappropriate medication use, as well as prescribing medications that do not lead to quick improvement, can prolong stays in emergency care, contributing to further stigma.

A vignette survey of resident doctors in France found that they maintain greater social distance from those diagnosed with psychiatric conditions and feel more uneasy when examining these patients in an emergency setting (3, 56). This is problematic, since in psychiatric emergencies careful examination is very important for differential diagnosis of both mental and organic diseases, especially for excluding delirium.

### Conclusions About Consequences of Stigma in Psychiatric Emergencies

As about causes, we found few studies on the consequences of stigma for mental illness, even less that referred to situations of psychiatric emergencies, the majority are non-systematic reviews and few cross-sectional data. Many studies (including a clinical trial) present such data indirectly. Types of consequences presented are only theories and should be investigated in future research.

## Stigma Reduction in Psychiatric Emergencies

Some measures were created to reduce stigma (57, 58), and most of the literature refers to programs already implemented in some countries. The following examples present some of the most frequent approaches to dealing with stigma, directly as a primary or secondary result to other activities: awareness, literacy programs, protest/advocacy, and social contact (38). Even so, there is little data on the benefit of measures to confront stigma. Corrigan et al. assessed protest/social activism, public education, and contact with persons with mental illness as potential methods (59). In the following section we will propose specific stigma-reduction interventions for psychiatric emergencies based on the results of relevant studies.

### Education

Educational measures for the stigma of mental illness modify stereotypes, replacing them with true information (e.g., the myth that the mentally ill are homicidal maniacs, given that homicide rates differ little between people with severe mental illness and the general population). Educational strategies include public service announcements, books, brochures, films, videos, websites, podcasts, virtual reality, and other audiovisual resources (59, 60). Education has been found to have positive effects on reducing stigma among adults and adolescents (59, 60). Although specific campaigns for psychiatric emergencies could not be found, considering that the stigma is similar, we speculate that educational interventions could increase respect for patients in crisis and reduce the reluctance to immediately seek assistance in a crisis.

Public programs on the concept, consequences and treatment of mental illness can alert the population to the suffering of mentally ill patients. Healthcare professionals should also be aware of their own health and seek help in case of burnout, insomnia, depression, or other mental problems. Emergency services should ensure the proper facilities and protocols to provide the most effective treatment possible, resulting in brief, effective, and comfortable stays.

More studies have been published on educational activities (particularly training) for health professionals, although they are for stigma in general, rather than emergencies. For example, a training program improved negative attitudes towards people with borderline personality disorder and those who get hurt (3). It has been proposed that interdisciplinary training in medical schools on mental illness and on the effectiveness of therapy can promote long-term benefits in health care and patient satisfaction (3). Many proposals aimed at changing the attitudes of medical students towards psychiatry focused on changing teaching curricula (43). We propose that teaching about psychiatric emergencies in medical school would not only improve the quality of care, but reduce stigma, as well.

Healthcare professionals should be trained to deal with major emergencies, such as suicidal behavior, psychomotor agitation, physical restraint, substance abuse disorders, psychotic breakdowns, mood episodes, anxiety attacks, eating disorders, personality disorder emergencies, and mental retardation. They must also be accustomed to treating different populations, such as different age groups (children, adolescents, adults, and older adults), both sexes, individuals with sexual preference and gender identity issues, the homeless, and those from different regions of the country (which can influence the expression of information and psychopathological assessment).

In the emergency room, care must be taken with physical restraint, which should only be used as a last resort to protect the patient in cases of psychomotor agitation (7, 61). Before physical restraint is considered, all other techniques should be employed, especially verbal de-escalation. Physical restriction presents significant risks (7, 62). Trauma from coercive techniques, which may seem a little coercive, can cause feelings of fear, humiliation, and helplessness, both for the patient and for the professionals (7, 31). Unexpected events can occur, such as orthopedic injury, dehydration, rhabdomyolysis, thrombosis, asphyxia, and even death (7, 62). Therefore, training, the use of appropriate techniques, and appropriate equipment should always be a priority. Restraint should never be seen as a mechanism of penalizing or coercion. The patient’s modesty must always be respected, and physical restraint must continue only for the shortest possible time.

Research has shown that health professionals can present pessimistic ideas about recovery. Poor skills and training can be related to stigmatization. This reported as a problem in the culture of some health services, where the team is often discouraged from talking or seeking help for psychological problems (4). Changing attitudes in emergency care settings, such as therapeutic pessimism, a lack of skills, and stigma-related workplace culture, are essential strategies. Health professionals who provide emergency care need support. Interventions could include clinical discussion groups and individual or group psychotherapy.

One institution has proposed a list of attitudes for reducing stigma (63); we have modified some of the statements for application in emergencies, either by healthcare professionals or the general population, when dealing with psychiatric emergencies:

- Find out about mental illness.
- The crisis and the emergency are fleeting and are only a small part of the disease and treatment
- Be aware of your attitudes and monitor your own critical thinking, which has been shaped by your education and society.
- Choose your words to positively influence the attitudes of others.
- Invest in guidance. Present positive facts and try to modify myths and stereotypes.
- Treat people with respect and always offer support and encouragement.
- Be aware of patients’ rights.

### Contact With the Mentally Ill

When people in the general population meet and interact with the mentally ill, their prejudice is likely to reduce (59, 64). Research has observed factors that appear to moderate the effect of contact (59, 65): one-on-one contact, which helps people discover similar interests and develop a friendship (59, 66), contact that includes a common goal (59, 67), and interaction with a person who moderately refutes prevailing stereotypes (59, 68). It was observed that contact has positive effects in reducing stigma in adults and adolescents. Contact was more effective in reducing stigma in adults and education was more effective in adolescents. Personal contact was more effective than video contact (59). Contact with the mentally ill is a powerful tool: patients and family members can share their experiences with emergencies, both in the community as well as with professionals during their training.

### Stigma Self-Management

Self-management encourages mentally ill patients to get the better of their identity and go beyond experiencing illness to find new personal motivations (9, 69, 70). Patients who have gone through emergency situations, have been under observation in emergency units or have been hospitalized should receive support to prevent embarrassment, as well as explanation that the situation was a complication of their disease and that the measures taken were necessary to protect them. But, there is still little evidence suffers the effectiveness of brief psychoeducation for patients with severe mental illness (71).

### Protest and Social Activism

Protesting highlights the injustice of many types of stigma and reprimands offenders for their stereotyping and prejudice (59). Some research has implied that campaigns to defeat discrimination can produce an unexpected recuperate effect, in which prejudices about a specific group remain unaffected or worsen (59, 72, 73). One set of studies (72) found that research participants who were asked to suppress pattern ideas about skinheads showed greater stereotype activation and increased social distance from this group (59).

### Legislative Reform and Advocacy

Advocacy for people with a mental illness is intended to prohibit discrimination, improve protection, and offer help with employment, education, and housing (70). Speech on human rights, such as that promoted by the United Nations Convention on the Rights of Persons with Disabilities (70), provided an important framework for legislative reform, including first generation protections against coercion and forced treatment and second generation protections that require governments and institutions to provide economic, social, and health support aimed at removing barriers to the social participation of mentally ill people (70, 74).

Such reforms are intended to ensure that people with mental illness use their rights and freedoms available in legislation and present systems for redressing complaints for unfair procedures. The World Health Organization defines advocacy as a way to broaden the notion of the importance of mental health issues and ensure that such a topic is on the governments’ agenda. Advocacy involves various strategies, including awareness raising, disseminating information, education, training, mutual help, counseling, mediating conflicts, defending the disadvantaged, and denouncing injustice. Advocacy is important because it should guide to direct changes in legislation and policy, as well as the development of services (9, 70).

Psychiatric emergencies must be included in such legislation. It is essential to have specific emergency response regulations in place, including minimum staff and facility criteria. Legislation allows for investment in hospital infrastructure prior to increased demand, when patients have difficulty receiving assistance in times of crisis.

### Conclusion About Stigma Reduction in Psychiatric Emergencies

There is also little work on intervention measures to reduce stigma. Mostly revisions. To facilitate, we divided into education, contact with the mentally ill, stigma self-management, protest and social activism, and legislative reform and advocacy. Only the first two had a trial or meta-analysis that assessed the effectiveness of the measures. Even so, not specific for psychiatric emergencies and without measures of effect size. Therefore, even stigma intervention measures for psychiatric emergencies are an open field for future research.

## Discussion

There is no doubt that the stigma devalues the mentally ill and the professionals and the health network set up to support them. Mental health programs and issues are not a priority or are of little importance to governments, which impairs the quality of treatment (when available) and impairs their quality of life, as well as the possibility of recovery (70). Mental health professionals and organizations are only now discovering advocacy and are more often seen as part of the problem, rather than the solution (70).

While this can undermine the possibility of leading change, mental health professionals can still be allied with anti-stigma initiatives and can advocate for adjustments in the treatment of the mentally ill. Mental health professionals in teaching roles can encourage students to learn about professional and political activism through contact techniques, encouraging the idea that recovery is not just symptom control (70).

Although there is little literature specifically on stigma in psychiatric emergencies, sufficient information exists for recognizing and preventing it. Emergency situations represent severe complications of mental illness that can involve social exposure, restraints, and more aggressive therapeutic measures. Health professionals must be alert and intervention measures, especially training, must be applied by healthcare institutions.

We would like to stress that studies on the subject have some limitations. Firstly, most are non-systematic reviews based on the opinion of the authors themselves. Second, in the few cross-sectional, longitudinal, and intervention studies, there is no specificity for situations of psychiatric emergencies. Third, most have evidence with little of poor quality. Therefore, it is still empirical, and somewhat open in research, with many gaps to be answered.

Future research will be able to evaluate the effectiveness of measures to combat stigma, focusing mainly on emergencies. Emergency training, involving both techniques and ethical aspects, as well as effective communication with the mentally ill during a crisis should be prioritized in public policy.

## Author Contributions

All authors participated in all stages of the article, including literature review, writing and approval of the final version.

## Conflict of Interest

The authors declare that the research was conducted in the absence of any commercial or financial relationships that could be construed as a potential conflict of interest.
